# Supplementary material for: Content-rich biological network constructed by mining PubMed abstracts
Source: BMC Bioinformatics. 2004 Oct 8;5:147. doi: 10.1186/1471-2105-5-147 (PMC528731; doi:10.1186/1471-2105-5-147)
Supplement: Additional File 2 — The original results of the above study (non-essential files are deleted to keep the file size under the limit set by BMC bioinformatics). [file 1471-2105-5-147-S2.bz2 › chilibotAdditionalFile2/dip05/600ID10092459E10979/html/PHES_PHET.html]

 


 **PHES** and **PHET** 
  
Found 54 abstracts in PubMed, retrieved 05.  
 

 What does Google say? 
 PDF only 
| .edu only 

---

**Interactive relationship** (e.g. stimulation, inhibition, etc)

**Neutral relationship**- Tn5luxAB was localized to a fragment of chromosomal DNA encoding the thrS, infC, rpmI, rplT,  **pheS**  and  **pheT**  genes, with Tn5 inserted in the 3 end of  **pheS** .  Ref: 12583892 FEMS Microbiol Lett, 2003

**Non-interactive relationship** (e.g. studied together, co-existance, homology, etc.)

- The extent of tRNA recognition at the level of binding by Thermus thermophilus phenylalanyl tRNA synthetase PheRS  [ **PHET** ]   [ **PHES** ] , one of the most complex class II synthetases, has been studied by independent measurements of the enzyme association with wild type and mutant tRNA Phe s as well as with non cognate tRNAs.  Ref: 12382236 J Mol Recognit< MedlineTA>J Mol Recognit, 2003
- The calculated binding energies of Phe analogues in PheRS  [ **PHET** ]   [ **PHES** ] , using HierDock, correlate well with the translational activities of the same analogues in Escherichia coli.  Ref: 12452720 J Am Chem Soc, 2002
- Starting with the crystal structure of Thermus thermophilus PheRS  [ **PHET** ]   [ **PHES** ]  without bound ligand, HierDock predicts the binding site of phenylalanine Phe within 1.1 A of that revealed by the crystal structure of PheRS  [ **PHET** ]   [ **PHES** ]  cocrystallized with Phe.  Ref: 12452720 J Am Chem Soc, 2002
- The dissociation constants for the eEF1A.GDP.tRNA and eEF1A.GDP.tRNAPhe.PheRS  [ **PHET** ]   [ **PHES** ]  complexes were found to be 20 nm and 9 nm, respectively.  Ref: 12354112 Eur J Biochem, 2002
- In this paper, we describe a computational protocol HierDock for predicting the relative energies of binding of phenylalanine analogues to phenylalanyl tRNA synthetase PheRS  [ **PHET** ]   [ **PHES** ] .  Ref: 12452720 J Am Chem Soc, 2002
- In particular, it is suggested that the monomeric PheRS  [ **PHET** ]   [ **PHES** ]  from the yeast mitochondrion is a chimera of the alpha and beta chains of the standard tetrameric protein.  Ref: 12572618 Mol Biol Evol, 1998
- We also revealed a direct interaction of PheRS  [ **PHET** ]   [ **PHES** ]  with eEF1A in the absence of tRNAPhe Kd = 21 nm.  Ref: 12354112 Eur J Biochem, 2002
- HierDock identifies p fluorophenylalanine and 3 thienylalanine as especially good substrates for PheRS  [ **PHET** ]   [ **PHES** ] , in agreement with experiment.  Ref: 12452720 J Am Chem Soc, 2002
- The highest affinity of T. thermophilus PheRS  [ **PHET** ]   [ **PHES** ]  for cognate tRNA, observed for synthetase tRNA complexes, results in 100 3000 fold binding discrimination against non cognate tRNAs.  Ref: 12382236 J Mol Recognit< MedlineTA>J Mol Recognit, 2002
- Here we present the data on association of the eEF1A.GDP.tRNA complex with phenylalanyl tRNA synthetase PheRS  [ **PHET** ]   [ **PHES** ] , e.g. the formation of the above quaternary complex detected by the gel retardation and surface plasmon resonance techniques.  Ref: 12354112 Eur J Biochem, 2002
- The data obtained, combined with kinetic data on aminoacylation, clearly show that PheRS  [ **PHET** ]   [ **PHES** ]  exhibits more tRNA selectivity at the level of binding than at the level of catalysis.  Ref: 12382236 J Mol Recognit< MedlineTA>J Mol Recognit, 2002
- A few backbone mediated contacts of PheRS  [ **PHET** ]   [ **PHES** ]  with the acceptor and anticodon stems revealed in the crystal structure do not contribute to tRNA Phe discrimination, their role being limited to stabilization of the complex.  Ref: 12382236 J Mol Recognit< MedlineTA>J Mol Recognit, 2002
